# Identification and analysis of immune-related transcriptome in Asian seabass *Lates calcarifer*

**DOI:** 10.1186/1471-2164-11-356

**Published:** 2010-06-04

**Authors:** Jun Hong Xia, Gen Hua Yue

**Affiliations:** 1 Molecular Population Genetics Group, Temasek Life Sciences Laboratory, National University of Singapore, 117604 Republic of Singapore

## Abstract

**Background:**

Fish diseases caused by pathogens are limiting their production and trade, affecting the economy generated by aquaculture. Innate immunity system is the first line of host defense in opposing pathogenic organisms or any other foreign material. For identification of immune-related genes in Asian seabass *Lates calcarifer*, an important marine foodfish species, we injected bacterial lipopolysaccharide (LPS), a commonly used elicitor of innate immune responses to eight individuals at the age of 35 days post-hatch and applied the suppression subtractive hybridization (SSH) technique to selectively amplify spleen cDNA of differentially expressed genes.

**Results:**

Sequencing and bioinformatic analysis of 3351 ESTs from two SSH libraries yielded 1692 unique transcripts. Of which, 618 transcripts were unknown/novel genes and the remaining 1074 were similar to 743 known genes and 105 unannotated mRNA sequences available in public databases. A total of 161 transcripts were classified to the category "response to stimulus" and 115 to "immune system process". We identified 25 significantly up-regulated genes (including 2 unknown transcripts) and 4 down-regulated genes associated with immune-related processes upon challenge with LPS. Quantitative real-time PCR confirmed the differential expression of these genes after LPS challenge.

**Conclusions:**

The present study identified 1692 unique transcripts upon LPS challenge for the first time in Asian seabass by using SSH, sequencing and bioinformatic analysis. Some of the identified transcripts are vertebrate homologues and others are hitherto unreported putative defence proteins. The obtained immune-related genes may allow for a better understanding of immunity in Asian seabass, carrying out detailed functional analysis of these genes and developing strategies for efficient immune protection against infections in Asian seabass.

## Background

Fish diseases caused by viruses, bacteria and parasites are recognized as a significant constraint on aquaculture production and trade hence affecting the economy seriously [1,2]. A global estimate of disease losses in aquaculture surpassed US$ 9 billion per year, which is about 15% of the value of world farmed fish and shellfish production [3]. Successful defence against pathogenic infection is dependent on the ability to detect the presence of the invading pathogen [4-6]. Teleost fish possess the elements of both the innate defence system and the acquired specific immune system [7]. However, the adaptive immune response in fish is less developed than that in higher vertebrates [5]. Therefore, innate immune system is quite important in fish and believed to be the first line of host defence in opposing pathogenic organisms or any other foreign material [4,7]. In aquaculture, the basic data on fish-pathogen interactions have been effectively applied for large scale vaccination to aid in the generation of robust and long-lasting immune responses [8,9]. However, the development of an effective vaccine is a complex process. The prerequisites for developing vaccines are the understanding of the basic epidemiology of diseases and the immune system of the target species and identifying the genes and pathways involved in transcript response of a fish upon infection [10,11].

Expressed sequence tags (ESTs) generated by cDNA cloning have proven to be a powerful and rapid tool for identifying genes [12-14]. ESTs also form the basis for subsequent microarray design, SNP detection and the placement of novel markers on genetic linkage maps [13,15-18]. Currently ESTs are available from a large number of fish species, such as European seabass [19], halibut [13], cod [20], trout [16], salmon [15], European and Japanese flounders [21] and catfish [17] which enabled the identification of immune-related genes in these species. However, screening for immune-related genes in EST databases using bioinformatic tools allows identification of only those genes that share sequence similarities with known immune-related proteins from other organisms [22], but misses novel genes related to immune responses. Suppression subtractive hybridization (SSH) [23] can be applied to identify differentially expressed genes in different tissues or conditions and thus is also a highly effective method for identifying novel genes related to important biological processes. This technique has been proven to be a suitable tool for identification of novel immune-related genes in a variety of teleost fish species, including flounder [21,24,25], rainbow trout [26], salmon [27-29], grouper [30], croaker [31], cod [32], sea bream [14,33], turbot [34], dogfish [35] and European seabass [36].

The Asian seabass *Lates calcarifer *distributed in the tropical and sub-tropical areas of Asia is an important marine foodfish species in Southeast Asia and Australia. This species has been cultured for more than 20 years in brackish-water ponds and in recent years in floating cages. The global annual production of Asian seabass was currently 400,000 metric tons according to FAO statistics [37,38]. In the past few years, we conducted a breeding program for Asian seabass [37] and developed a number of genomic tools such as microsatellites [39], SNPs in genes [40], linkage maps [37], BAC and cDNA libraries [40,41] to facilitate the selective breeding program. Massive mortalities caused by bacterial or viral infections in intensive aquaculture production have caused serious economic losses in Asian seabass aquaculture. Some kinds of bacteria, such as *Cytophaga johnsoniae *and *Streptococcus iniae *have currently been isolated from sick seabass in Singapore [42], Thailand [43] and Australia [44,45]. In order to shield the aquaculture loss by pathogenic diseases, Asian seabass aquaculture urgently requires effective disease prevention strategies. Although some studies had shown that Asian seabass exhibited strong immune responses against bacteria based on the antibody activities in sera [46], little information on host--pathogen interaction during infection with pathogenic microorganisms is available for this species.

Immune response can be experimentally stimulated by bacterial lipopolysaccharides (LPS) [47-49]. cDNA libraries of liver, kidney and spleen have been proven to be an excellent source of genetic information concerning immune function in fish [13]. The aim of this study was to identify and analyze immune-response genes in Asian seabass by challenging individuals with LPS, the SSH technology and bioinformatic tools.

## Results

### SSH efficiency

The efficiency of subtraction can be estimated with PCR analysis by comparing the abundance of housekeeping genes and differentially expressed genes before and after subtraction (Clontech manual). In this experiment, an immune-related gene (the immunoglobulin heavy constant mu: IGHM) and a house-keeping gene (ί-actin) were used to examine the efficiency of subtraction. Quantitative real-time PCR analysis of IGHM and ί-actin revealed that SSH efficiency was 3.4 fold for the forward subtractive library and 4.7 fold for the reverse subtractive library.

### Generation of subtractive cDNA libraries and assembly of ESTs

Two subtractive cDNA libraries, a forward subtractive library (genes expected to be up-regulated in response to immune challenge in this library) and a reverse subtractive library (genes expected to be down-regulated in response to immune challenge) were constructed using subtractive cDNA from spleen of Asian seabass sampled at 24 hour post challenge with LPS and control samples. A total of 1527 and 1824 randomly picked clones for the forward subtractive library and the reverse subtractive library, respectively, were sequenced. After trimming of end and vector sequences and eliminating sequences with low quality and/or shorter than 100 bases, a total of 2887 high quality sequences were obtained. Among the 2887 high quality sequences 1168 were derived from the forward subtractive library whereas 1719 from the reverse subtractive library (Table 1).

**Table 1:** EST sequences and assembly statistics for two suppression subtractive hybridization libraries constructed from spleen cDNA of Asian seabass

	Forward subtractive library	Reverse subtractive library	Sequences combined
Total number of sequences	1527	1824	3351
Clean sequences (>100 bp)	1168	1719	2887
Low quality sequences	359	105	464
Total number of unique sequences	761	1093	1692
Singlets	588	797	1207
Contigs	173	296	485
2 ESTs	118	196	292
3-4 ESTs	41	72	130
≥5 ESTs	14	28	63
SNP-contained contigs	--	--	303
Microsatellite -contained unique sequences (≥8 repeats)	--	--	20

After assembly and clustering, 1692 unique transcripts consisting of 1207 singletons and 485 contigs (Table 1) were obtained. Among the 485 contigs, 290 (60.2%) contained 2 ESTs, 130 (26.8%) had 3-4 ESTs and 63 (13.0%) consisted of 5 or more sequences. The largest contig was formed by 97 ESTs.

### Annotation of ESTs

Blast searches against known sequences in public databases using the programs BLASTx and BLASTn revealed that 1074 unique transcripts were similar to 743 known genes and 105 unannotated mRNA sequences with high confidence (E value < 10^-4^) in the database. The remaining 618 unique transcripts were potentially novel sequences or UTRs of known genes. Three hundred and thirty-one of these known genes and unannotated mRNA sequences were represented by multiple sequences (see Additional file 1). Among the known genes and mRNA sequences represented by EST clones, 542 were found in the reverse subtractive library and 467 were found in the forward subtractive library, respectively. Of which, 161 were present in both libraries. The percentage (~9%) of unique transcripts presented both in reverse and forward subtractive libraries was slightly higher than that in some previous studies (3-5.95%), such as on grouper *Epinephelus coioides *[50]. This might be related to the nature of SSH technique used in this study. Although differentially expressed genes were enriched significantly with the approach, some of the unwanted genes might not have been eliminated completely in the libraries. When more clones of the SSH libraries were sequenced, as in our study, more rare transcripts would be found in both of the forward and reverse libraries. This should increase the percentage of unique transcripts that are present both in reverse and forward subtractive libraries.

The globin gene family was among the predominant EST sequence clusters in both libraries. Of the 2887 EST sequences, 13.8% (397) matched the globin family (hbaa1, ba1, hbb, Hba-x), 5.7% (165) were ribosomal proteins (12 S, 16 S, 18 S, 40 S and 60 s), 1.6% (47) were initiation factors and 1.2% (34) belonged to elongation factors (Additional file 1). These results probably reflect that the fish were in an active growth stage. Interestingly, the expression of the globin gene family (p < 0.0001) and elongation factors (*p *= 0.0023) were significantly differentiated between two libraries, indicating some globin proteins (up-regulated upon LPS challenge) and elongation factors (down-regulated upon LPS challenge) might play important roles in host defence.

The complete results of BLAST searches of the Asian seabass ESTs from the forward subtractive and reverse subtractive libraries are summarized in Additional file 1.

### Functional classification

Available bioinformatic tools, such as the Gene Ontology (GO) GO Slimmer and Kyoto Encyclopedia of Genes and Genomes (KEGG) provide useful information to analyze the functional profile of annotated genes [51]. A total of 743 annotated genes were used in functional classification (Additional file 1). According to GO Slimmer results, genes encoding for proteins associated with cellular process, metabolic process and biology regulation in category of biological processes (Additional file 2) were the three largest annotated subcategories. The second largest group was genes encoding products related to regulation of biological process, response to stimulus and developmental process in both of the forward subtractive library and the reverse subtractive library. The remaining genes encoded products were involved in many other diverse biological processes. With respect to cellular components (Additional file 3), we observed that a large proportion of sequences were classified into cell, cell part and organelle followed by organelle part, macromolecular complex, extracellular region and membrane-enclosed lumen in the forward and the reverse subtractive libraries. As expected, a remarkably high proportion of annotated sequences were categorized as binding and catalytic activity followed by transcription regulator activity and transporter activity in both of the libraries (Additional file 4). Analysis of GO categories showed that the functional distribution of the genes of the two libraries in the three categories was similar (*P *> 0.05). The detailed information of functional classification is shown in Additional file 5 and 6.

KO (KEGG Orthology) is a pathway-based classification of orthologous gene groups. Four hundred and sixty seven of the 1692 unique transcripts with KO assignments were composed of 402 unique genes. Of which, 257 unique genes belonged to 160 KEGG pathways. Interestingly, 52 annotated genes were found in 10 immune-related pathways, including complement and coagulation cascades (13 hits), chemokine signaling pathway (12 hits), antigen processing and presentation (10 hits), Toll-like receptor signaling pathway (9 hits), leukocyte transendothelial migration (8 hits), T cell receptor signaling pathway (7 hits), hematopoietic cell lineage (6 hits), B cell receptor signaling pathway (5 hits), natural killer cell mediated cytotoxicity (4 hits) and Fc epsilon RI signaling pathway (3 hits). Most of the annotated genes were associated with cellular process (213 hits) and diseases (171 hits) and metabolism (167 hits), followed by environmental (79 hits) and genetic (70 hits) information processing. Our data suggest that several important immune processes were likely to be affected by the LPS challenge.

### Immune-related genes and differentially expressed genes in subtractive libraries

Genes in the GO subcategories 'response to stimulus' and 'immune system process' are likely to be involved in the interaction of bacteria with its host. We identified a total of 161 genes (78 in the forward subtractive library and 111 in the reverse subtractive library) with response to the LPS challenge, whereas only 28 genes were shared between two libraries. These genes were mainly associated with response to stress (81), response to chemical stimulus (80), response to external stimulus (48), cellular response to stimulus (40), regulation of response to stimulus (35) and immune response (36). Most of the responsive genes were under represented in the library with only one copy in each library. Although the percentage of unique genes responding to stimulus contained in both libraries was not significant, the actual number of the EST clones representing these genes (396 in the forward subtractive library and 384 in the reverse subtractive library) was quite different between two libraries (*P *< 0.0001). In addition, 13.3% of the EST clones (383) matched the genes in immune system process (115 unique genes) based on GO term and KAAS data. The EST clones of immune genes were found statistically different between two libraries (*P *< 0.0001). Majority of the immune and stress-related EST sequences identified in this study (Additional file 5 and 6) were reported for the first time in the Asian seabass.

Based on the number of homologous ESTs in the two libraries, Fisher's exact test found a significant increase in abundance for a total of 25 genes (including 2 unknown genes) and a significant decrease in abundance for 4 genes upon challenge with LPS, suggesting a strong transcriptional regulation upon LPS challenge (Table 2). Homologues of some immune-related genes such as LY6 D and EEF2L were found exclusively in one library, and some others such as C1QL4L and HMOX1 were found highly expressed not only in one library but also in another library at a much lower frequency. Only 6 (IGH-1B, C3, FGG, LGMN, B2 M and HMOX1) of the 29 genes that differentially expressed in spleen of the Asian seabass upon challenge with LPS were classified as immune response-related genes in the gene ontology database [52]. However, 15 of the remaining 23 differentially expressed genes could be identified as immune response-related genes in InnateDB non-redundant list [53] except BA1, AGC1, HDR, HBAA1, RPS7, EEF and two unknown genes.

**Table 2:** Immune response-related genes differentially expressed in Asian seabass at 24 h post challenge by bacterial lipopolysaccharides (*P *< 0.05)

Cluster ID	Gene ID and description	Count-Up	Count-Down	P-value
**Up-regulated genes**				
T1	BA1; ba1 globin (*Danio rerio *)	162	141	0.0000
T2	HBAA1; hemoglobin alpha adult-1 (*Danio rerio *)	48	36	0.0021
T4	C1QL4L; complement component 1, q subcomponent-like 4 like (*Danio rerio *)	25	1	0.0000
T23	LY6D; lymphocyte antigen 6 complex, locus D (*Mus musculus *)	9	0	0.0003
T32	IGH-1B; immunoglobulin heavy chain 1b (*Homo sapiens*)	7	1	0.0091
T33	C3; complement component 3 (*Mus musculus*)	8	0	0.0007
T34	SAA3; serum amyloid A 3 (*Mus musculus*)	8	0	0.0007
T44	APOE; apolipoprotein E (*Canis lupus*)	7	0	0.0016
T45	NCAN; neurocan (*Mus musculus*)	7	0	0.0016
T59	RPS7; 40 S ribosomal protein S7 (*Homo sapiens*)	5	1	0.0429
T60	RRT15; uncharacterized protein YLR162W-A (*Saccharomyces cerevisiae*)	5	1	0.0429
T61	AGC1; aggrecan 1(*Danio rerio*)	6	0	0.0044
T82	FGG; fibrinogen, gamma polypeptide (*Rattus norvegicus*)	5	0	0.0108
T83	HDR; hematopoietic death receptor (*Danio rerio*)	5	0	0.0108
T84	LCP1; lymphocyte cytosolic protein 1 (*Mus musculus*)	5	0	0.0108
T85	LGMN; legumain (*Danio rerio*)	5	0	0.0108
T127	APO; apolipoprotein (*Lates calcarifer*)	4	0	0.0267
T128	B2M; beta-2 microglobulin (*Mus musculus*)	4	0	0.0267
T129	CFL2; cofilin-2 (*Gallus gallus*)	4	0	0.0267
T130	GUK1;guanylate kinase (*Homo sapiens*)	4	0	0.0267
T131	mt-ND5; NADH-ubiquinone oxidoreductase chain 5 (*Homo sapiens*)	4	0	0.0267
T132	TKTL2; transketolase-like protein 2 (*Rattus norvegicus*)	4	0	0.0267
T133	WBP2; WW domain-binding protein 2 (*Homo sapiens*)	4	0	0.0267
Cg0105	Unknown mRNA sequence	4	0	0.0267
Cg0526	Unknown mRNA sequence	4	0	0.0267
**Down-regulated genes**				
T6	HMOX1; heme oxygenase (decycling) 1 (*Danio rerio *)	1	19	0.0008
T17	EEF2L; eukaryotic translation elongation factor 2, like (*Danio rerio*)	0	9	0.0134
T35	DBPHT; DNA binding protein with his-thr domain (*Mus musculus*)	0	7	0.0463
T36	EEF; eukaryotic translation elongation factor (*Siniperca chuatsi*)	0	7	0.0463

Count-Up, EST count of a given gene in the forward subtractive library; EST count in the reverse subtractive library. *P *< 0.05 indicates statistically significant.

By the GO classification scheme, the significantly differentially expressed genes were divided into subsets based on their functions. Among the genes with increased abundance, 6 major groups can be identified, including binding (IGH-1B, C3, APOE, WBP2, B2 M, LY6 D, CFL2, LCP1, FGG, RPS7), catalytic activity (LGMN, mt-ND5, GUK1), transporter activity (APOE and AGC1), enzyme regulator (APOE, C1QL4L), antioxidant activity (APOE) and structural molecule activity (LCP1 and RPS7) and with no hit for the remaining 10 genes (BA1, SAA3, NCAN, HDR, APO, TKTL2, HBAA1, RRT15 and 2 unknown genes). For the abundance-decreased genes, GO annotation reveals their involvement in several subcategories of molecular function including binding (HMOX1, DBPHT1), catalytic activity (HMOX1) and molecular transducer activity (HMOX1) and no assignment for two remaining genes (EEF, EEF2L).

### ESTs containing microsatellites and SNPs

Screening of all EST sequences for microsatellites (2-6 bp) identified 18 microsatellites with ≥8 repeats (Additional file 7). Out of these 18 microsatellites, 6 had significant hits with known genes (CCL25, KRT13, C4BP, CAHZ, LSM4 and EIF3H) by BLAST (e-value ≤ 1e^-4^). Twelve (67%) of the microsatellites were dinucleotide and 4 were trinucleotide (22%), while only 2 tetranucleotide (11%) were found. Because 8 individuals were used in library construction in this study and around 3000 EST sequences were available, it was possible to identify high quality SNPs. Three hundred and three of the 485 contigs (62.5%) contained SNPs, indicating that a very high polymorphism exists in genome of the Asian seabass among individuals.

### Validation of subtractive library data by quantitative RT-PCR (qRT-PCR) and expression profiles of genes in response to LPS challenges

**Figure 1:**
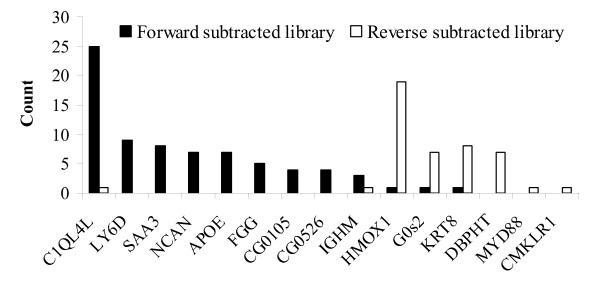
**The EST counts in two libraries for selected genes used in quantitative RT-PCR analysis**.

**Figure 2:**
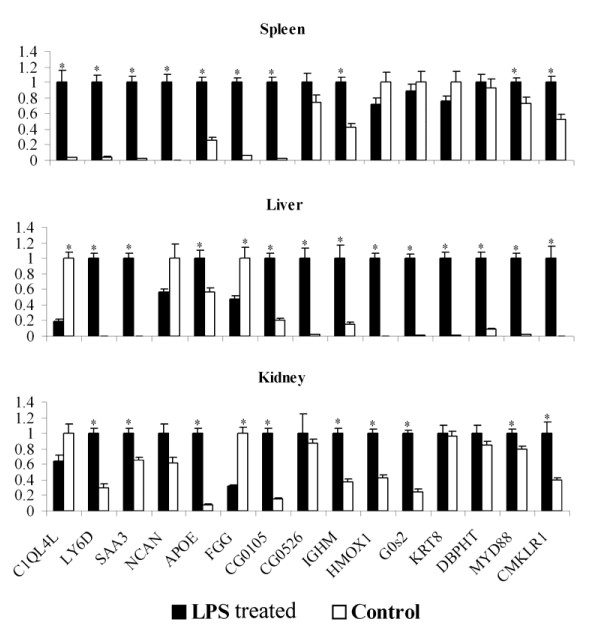
**Analysis of gene expression in spleen, liver and kidney of Asian seabass by quantitative RT-PCR**. Expression levels in three tissues were normalized using EF1A gene as the reference gene. Normalized fold expression data (mean ± s.e.) represented the average of three independent experiments. A star indicated that the expression of the corresponding gene was significantly different (*P *< 0.01) between LPS treated seabass and control.

To validate the subtractive library data, qRT-PCR was performed on 13 randomly selected annotated genes and 2 unknown gene sequences. Of which, 9 genes were considered to be up-regulated and 6 genes were presumably down-regulated in spleen upon LPS challenge, based on the differences of homologous EST counts in both libraries (Fig. 1). The expression levels for these genes were evaluated on the spleen RNA samples at 24 h post challenge with LPS and in the control group. The expression levels were normalized with a house-keeping gene, elongation factor 1-alpha (EF1A) and are presented as in Fig. 2. In well agreement with the EST data, all genes with higher counts in the forward subtractive library tested in the qRT-PCR assay showed clear induction in the spleen upon LPS challenge and three (HMOX1, G0s2 and KRT8) of the remaining genes with higher counts in the reverse subtractive library showed clear suppression after injection with LPS. However, the expression levels for the remaining genes MYD88 and CMKLR1 in two groups were significantly different from the EST counts in two libraries. This inconsistency might be caused by the random sampling errors in sequencing since their expressions were very low in spleen with only one copy in reverse subtractive library (Fig. 1).

In addition to the quantitative analysis of the expressions of these genes in spleen by qRT-PCR, expressions of these genes in liver and kidney in experimental (at 24 h post challenge with LPS) and control groups were analyzed using qRT-PCR to add information about the spatial expression of these genes. As shown in Fig. 2, it was surprising that all of the 6 genes down-regulated in spleen were highly up-regulated in liver and 4 of which were significantly up-regulated in kidney, except gene KRT8 and DBPHT. But for the 9 genes up-regulated in spleen, only 6 were significantly up-regulated in liver and 5 were significantly up-regulated in kidney as detected by qRT-PCR assay.

## Discussion

### Discovery of novel ESTs from the subtractive cDNA libraries of Asian seabass spleen

SSH is an effective method to study functional expression levels of specific transcripts in various cells and tissues [54]. This study identified a total of 1692 unique transcripts, of which 1074 were similar to 743 known genes and 105 unannotated mRNA available in public databases and the remaining 618 unique transcripts were potentially novel sequences or UTRs of known genes. Some of these genes might play important roles in host-pathogen interaction during infection. Future functional studies of these genes could improve our understanding of innate defence system of fish against pathogenic infection.

Currently, total EST collection for the Asian seabass in NCBI EST database [55] is approaching to 5637 (dated at April 24, 2010) and all of these data originated from a brain cDNA library [56]. The addition of 1692 unique transcripts from Asian seabass to the existing database would not only make a contribution to functional genomic studies, but also help the annotation of genome and the comparative analysis of gene expression profiles in the near future.

### Genes involved in innate immune responses

Fish represents the earliest class of vertebrates possessing the elements of both innate and acquired immunity [57,58]. The soluble mediators released by fish immune cells can regulate inflammatory responses and have a fully functional complement system and unique receptors that recognize pathogens [59,60]. We identified a total of 161 annotated genes (19%) in response to stimulus and 115 genes (13.6%) matching genes in immune system process. It was much higher than those reported in European seabass (*Dicentrarchus labrax*) with only 79 genes (6%) categorized to the GO category "immune system process" after infected with *V. anguillarum *[19] and with 8.7% of the ESTs showing significant similarities with immune genes after Nodavirus infection [36]. The majority of immune and stimulus-related EST sequences detected in this study were reported for the first time in the Asian seabass. Of which, some homologous genes found in this study were previously reported to play an important role in the innate immune response of vertebrate animals upon bacterial infection, e.g., Hepcidin [61]. Based on our EST data, we identified hepcidin-1 (hep-1) gene in the Asian seabass. The cDNA sequence with a length of 710 bp consisted of the whole ORF and partials of the UTRs for Hepcidin and encoded a peptide of 89 amino acids with a molecular weight of 9914 Daltons. This represents the first antimicrobial peptide gene discovered in the Asian seabass.

Many pro-inflammatory cytokines such as tumor necrosis factor (TNF)-like, interleukin-1 (IL-1) and IL-1-like receptors have been identified in a number of fish species [59]. In our study some pro-inflammatory cytokines and related receptors have also been identified, such as TNF receptor super family member 14 (TNFRSF14), IL-31 receptor A (IL31RA), chemokine receptor-like 1 (CMKLR1), chemokine (C-X-C motif) receptor 3 (CXCR3), chemokine (C-C motif) receptor 7 (CCR7) and chemokine (C-C motif) ligand 25 (CCL25). Complement components are also known to play a role in linking innate and adaptive immunity. A number of complement and complement receptor homologs such as C1R, C1 inhibitor, C2, C4, C3, C5, C6, C7, C8 and C9 have also been identified from a variety of fish species [59,62-64]. From our EST data, we identified complement component C1 (c1ql4l, C1QC, C1qb), C2 and CR2, C3 and C3B and complement factor (CFD and CFP). To our knowledge, the complement receptor CR2 (CD21) has not been previously reported in fish.

Lectins are also known to play an important role in the innate immunity of fish [59]. Two C-type lectins (TCL-1 and TCL-2) have been identified in rainbow trout [65,66] and C-type lectin receptors have also been identified in two species of cichlid fish, *Paralabidochromis chilotes *and *Oreochromis niloticus *[67]. We identified C-type lectin domain family 4 member C (CLEC4C), C-type lectin domain family 4 member E (CLEC4E) and a kind of F-type lectins (fucolectins) in our libraries. In addition, the Toll-like receptors (TLR) were reported to play a critical role in innate immunity against fungal and bacterial infections by initiating intracellular signal transduction that results in the expression of genes involved in inflammation, antiviral responses and maturation of dendritic cells [7,68]. We found at least 9 genes were involved in Toll-like receptor signaling pathway such as phosphoinositide-3-kinase, regulatory subunit (PIK3R), tumor necrosis factor receptor superfamily, member 5 (TNFRSF5, CD40), proto-oncogene protein c-fos (FOS) and Ras-related C3 botulinum toxin substrate 1 (RAC1).

### Differentially expressed genes associated with immune processes

A number of similarities in immune response exist between mammals and fish [59,69]. Analysis of the immune system of the zebrafish revealed a fully developed adaptive and innate immune system showing notable similarities to the mammalian equivalent [70]. By comparative analysis of ESTs, many conserved genes responded upon the bacterial infection were revealed between Asian seabass and mammals. For example, apolipoprotein E (ApoE) was found to work as an immune modulator in humans [71], European seabass, carp and medaka [19]. In this study we also detected a substantial increase in the expression of this gene upon LPS challenge. Based on the approximate expression patterns inferred from spleen EST sources of mammals [55], we concluded that more than half of the differentially expressed genes detected in this study were also found to be highly expressed in the spleen of mammals. Some of the genes detected in this study have highly homologous counterparts of well-known mammalian spleen genes such as BA1, EEF2L and C1QL4L, being a homologue of Hbb-b1, EEF2 and C1QA, respectively. All together, these data indicate that there are many conserved features in expression and function of genes in spleen between fish and mammals.

Our data also suggest that 8 (HDR, BA1, AGC1, HBAA1, RPS7, EEF and two unknown genes) out of the 29 significantly differentially expressed transcripts could not be identified as immune response-related genes in current public databases. The hematopoietic death receptor (ZH-DR) and the BA1 globin gene were highly differentially expressed upon LPS challenge. ZH-DR was the first TNFR family member to be identified in zebrafish and specific to haematopoietic tissue [72]. However, little information of the function of this gene in innate immune response is available currently. It was reported that death receptors belong to the TNF (tumor necrosis factor)/NGF (nerve growth factor) receptor superfamily and contribute to regulation of the adaptive immune response [73]. Some reports also suggested that hemoglobin can be induced in macrophages stimulated with LPS and interferon-γ that synergistically increases the release of pro-inflammatory cytokines from the innate immune system in response to LPS [74,75]. Our result suggested that similar to other DR and hemoglobin genes, ZH-DR and BA1 globin gene might have potential immune regulatory functions in response to bacterial infection in fish. Alternatively, mammalian heme oxygenase 1 (HMOX1) was up-regulated strongly during stress and following pathogen entry [76,77]. To our surprise, HMOX1 was found to be significant down-regulated in spleen of seabass during the acute inflammatory response in this study. Another study of Atlantic salmon (*Salmo salar *L.) also showed that HMOX1 was down-regulated in spleen to infection with the salmon louse (*Lepeophtheirus salmonis*) at 22 day post infection [78]. Taken together, these data suggest that some differences might exist in immune-mediated inflammatory responses during host--pathogen interaction between fish and mammal.

### Putative markers in innate immunity

The development of fish comparative immunology has been hampered by the lack of specific markers for immunoregulatory peptides [79]. In this study at least 29 genes that significantly responded on the challenge of LPS were detected and almost all of the genes could be classified as immune response-related genes. In addition, a reliable predictive of changes in gene expression with the EST data in two libraries was confirmed by qPCR experiments; our study also showed that the expression of these genes was highly conserved between fish and mammals. Therefore, these significantly differentially expressed genes might be considered as candidate markers of bacterial infections in spleen of the Asian seabass. These genes provide the basis for further research into the identification of specific markers for immuno-regulatory peptides and for understanding the immune response of the Asian seabass.

### Microsatellites and SNPs in immune-related ESTs

In this study, we identified 18 microsatellites and over 300 SNPs in immune-related ESTs. These markers could be used for many genetic studies [80,81], such as linkage and QTL mapping [37], examining population dynamics [82,83], analyzing pedigree [84], managing genetic diversity of broodstock [85] and conducting comparative genome analysis [86,87].

## Conclusions

By challenging Asian seabass with bacterial lipopolysaccharide (LPS) and using suppression subtractive hybridization (SSH) technique and bioinformatic tools, we identified a large number of potential immune-related genes. Some of which are vertebrate homologues and others are hitherto unreported putative defence proteins. These genes will supply us a solid basis for a better understanding of immunity in Asian seabass, for conducting detailed functional analysis of these genes and for developing effective strategies for immune protection against infections in the Asian seabass.

## Methods

### Fish

Around 100 small Asian seabass at the age of 15 dph were transported from a commercial fish farm to TLL animal house. The fish were maintained in a large tank containing 500 L seawater at 25°C for acclimatization of 3 weeks. Fish were fed twice daily with palliated feed.

### Challenging with LPS and sampling

One day prior to challenge, 16 healthy fish individuals of average weight of 5 g were transferred to two smaller tanks holding 10 L of sea water. For 8 fishes in tank 1, each fish was injected intra-peritoneally with 0.1 ml of 2 mg/ml of *Escherichia coli *LPS (Sigma-Aldrich, Saint Louis, USA) by dilution with phosphate buffered saline (PBS) at RT. In tank 2 (control), each of the 8 fishes received an intra-peritoneal injection of 0.1 ml of PBS. Just before injection and sampling, the fishes were anaesthetized using AQUI-S^® ^with a concentration of 15 mg/L (AQUI-S New Zealand Ltd, Lower Hutt, New Zealand). Eight fishes from each tank were sacrificed at 24 h post challenge. Tissues including spleen, kidney and liver were taken from every fish of each tank and kept in Trizol reagent (Invitrogen, Carlsbad, USA) at -80°C until use.

### RNA isolation

Total RNA from spleen was isolated using the TRIZOL kit (Invitrogen, Carlsbad, USA) according to the manufacturer's instructions. Purification of mRNA from total RNA was carried out using Oligotex mRNA Midi Kit (Qiagen, Valencia, USA). The concentration and purity of mRNA were measured using a NanoDrop Spectrophotometer, ND-1000 (NanoDrop Technologies, Wilmington, USA).

### Subtractive library construction and sequencing of clones

cDNA suppression subtractive libraries enriched for differentially expressed genes were constructed using PCR-Select cDNA subtraction kit (Clontech, Mountain view, USA) according to the manufacturer's protocol. Isolation of pure poly A+ mRNA from total RNA was performed using Oligotex^® ^mRNA Mini Kit (Qiagen, Valencia, USA) according to the manufacturer's protocol. The resulting mRNA from 8 LPS-challenged fishes and the mRNA from 8 PBS-treated fishes (control group) was mixed in equal quantity separately. The forward and reverse subtraction experiments of 1 μg of the mixed spleen mRNA were performed for both samples. The efficiency of subtraction was estimated by comparing the abundance of known cDNA (ί-actin and IGHM) before and after subtraction with quantitative RT-PCR. The PCR products of subtractive cDNA were directly inserted into a pGEM-T vector (Promega, Madison, USA) and transformed into *E. coli *strain XL-1 (Stratagene, La Jolla, CA) to make two subtractive cDNA libraries, a forward subtractive library (genes expected to be up-regulated in response to immune challenge in this library) and a reverse subtractive library (genes expected to be down-regulated in response to immune challenge). A total of 3551 randomly picked clones from two libraries were sequenced in both directions with M13 forward and M13 reverse primers using BigDye chemicals and ABI 3730 × l Genetic Analyzer (Applied Biosystems, Foster city, CA).

### Sequence analysis and functional annotation

Base calling from chromatogram traces and trimming of vector and adaptor sequences and low-quality regions from EST sequences were performed by using commercial software Sequencher 4.9 (Gene Codes, Ann arbor, MI, USA). Then, high quality ESTs (≥100 bp) of both forward and reverse subtractive libraries were used to form contigs. These contigs were manually revised to detect possible errors. Singletons and consensus sequences of each contig were referred to as unique sequences and were compared against the gene ontology database[52] using BLASTx. The remaining sequences without significant assignments were compared against the NCBI database [55] using BLASTn. The significant UniGene information (e value cut off was ≤1e^-4^) of the query set of unique sequences subsequently were mapped into several level 1 subcategories of the three broad categories of 'cellular component', 'molecular function' and 'biological process' respectively, with software GO Slimmer [52]. The KO (KEGG Orthology) assignments and KEGG pathway reconstruction were performed in KAAS (Automatic Annotation Server Ver. 1.6a) [88]. Antimicrobial peptides were identified based on the Antimicrobial Peptide Database (APD) [89] and the assignments for the 29 differentially expressed genes were also carried out in InnateDB non-redundant list [53]. All of the EST sequences were submitted in GenBank with accession nos GT219120-GT222006.

### Mining of microsatellites and SNPs

All of the unique sequences were searched for microsatellites using the program Tandem Repeats Finder (ver. 4.00) [90]. The repeat units were set to 2-10 and other parameters were set to default. The microsatellite-containing ESTs (with ≥8 repeat number) were identified as candidates for future marker development. Single nucleotide polymorphisms (SNPs) in contigs were detected manually.

### Statistical analysis

A web tool IDEG6 was used for detection of differentially expressed genes between two libraries using Fisher exact test [91]. The *P *value of less than 0.05 was considered statistically significant for these analyses. For testing the null hypothesis that the two means of gene expressions between LPS treated seabass and control were equal, a two-tailed T-test was performed by using the web calculator 'Independent groups T-TEST for means calculator' [92]. A confidence level of 99% was used in the test.

### Analysis of gene expression by quantitative real-time PCR

Spleen, liver and kidney samples were collected from three seabass individuals challenged with LPS and three individuals from the control group (only injected with PBS, see details in the section "Challenging with LPS and sampling") at 24 h post challenges. Total RNA was isolated using the TRIZOL kit (Invitrogen, Carlsbad, USA) according to the manufacturer's instructions. After DNase treatment with DNase I recombinant (Roche, Branchburg, USA) and purified by phenol-chroform, around 1 μg aliquot of the DNase-treated total RNA were reverse transcribed to cDNA by M-MLV reverse transcriptase (Promega, Madison, USA) with 0.67 μM poly dT as RT primer in 15 μl volume following the manufacturer's protocol. The reaction mixture of the RNA template and RT primer was heated at 70°C for 5 min to denature the RNA and then incubated on ice for 5 min. The remaining reagents were added as specified in the thermoscript protocol and the reaction proceeded for 1 hour at 42°C. Finally, the reverse transcriptase was inactivated by incubation at 70°C for 15 min.

For the analysis of expression patterns, the resulting single strand cDNA were 10 times diluted and assayed as DNA template by real-time PCR using primers (see Additional file 8) for 15 different genes. The EF1A gene was used as control (Fig. 2) as suggested by Olsvik et al. [93] and Jorgensen et al. [94]. PCR was performed with the iQ SYBR Green Supermix (Bio-Rad, Hercules, CA, USA) in an iQ™ 5 Real Time PCR Detection Systems (Bio-Rad, Hercules, CA, USA). Briefly, the 25 μl of reaction including 12.5 μl SYBR Green Supermix, 200 nmol each primer and 1 μl diluted cDNA was initially denaturated at 95°C for 3 min, then amplified for 40 cycles (95°C, 5 s, 55 or 60°C, 10 s and 72°C, 20 s). PCR was performed in triplicates. Values shown in Fig. 2 were the average of triplicate real-time PCR reactions, normalized to EF1A gene expression. A qRT-PCR MIQE checklist is shown in Additional file 9.

All procedures conducted with Asian seabass fish were adhered to animal care guidelines (Guidelines on the Care and Use of Animals for Scientific Purposes) as outlined by the National Advisory Committee For Laboratory Animal Research in Singapore. An approval is not required as both authors have the certificate of responsible care and use of laboratory animals. The remaining fish were used for other experiments, e.g., construction of cDNA libraries, isolation and identification of microRNAs.

## Abbreviations

SNP: single-nucleotide polymorphism; EST: expressed sequence tag; SSH: suppression subtractive hybridization; LPS: bacterial lipopolysaccharides; UTR: untranslated region; GO: gene ontology; KEGG: kyoto encyclopedia of genes and genomes; qRT-PCR: quantitative real-time polymerase chain reaction and MIQE: Minimum Information for Publication of Quantitative Real-Time PCR Experiments.

## Authors' contributions

GHY initiated and overviewed the Asian seabass project and finalized the manuscript. JHX designed, carried out the study and drafted of the manuscript. Both authors have read and approved the final manuscript.

## Supplementary Material

Additional file 1 **Table S1**. Summary of BLAST searches of the Asian seabass ESTs from subtractive libraries. Click here for file

Additional file 2 **Fig. S1**. Classification of 743 annotated genes from Asian seabass in subcategories of biological process following GO. Click here for file

Additional file 3 **Fig. S2**. Classification of 743 annotated genes from Asian seabass in subcategories of cellular component following GO. Click here for file

Additional file 4 **Fig. S3**. Classification of 743 annotated genes from Asian seabass in subcategories of molecular function following GO. Click here for file

Additional file 5 **Table S2**. Functional classification of the Asian seabass genes from the reverse subtracted library following GO. Click here for file

Additional file 6 **Table S3**. Functional classification of the Asian seabass genes from the forward subtracted library following GO. Click here for file

Additional file 7 **Table S4**. Summary of unique sequences containing microsatellites in two subtractive libraries of Asian seabass. Click here for file

Additional file 8 **Table S5**. Primers used in the analysis of gene expression in spleen, liver and kidney of Asian seabass by quantitative RT-PCR. Click here for file

Additional file 9 **Lca-SSH qRT-PCR MIQE checklist**. The qRT-PCR experiment report. Click here for file
