# Supplementary material for: Identification and analysis of immune-related transcriptome in Asian seabass Lates calcarifer
Source: BMC Genomics. 2010 Jun 4;11:356. doi: 10.1186/1471-2164-11-356 (PMC2893601; doi:10.1186/1471-2164-11-356)
Supplement: Additional file 2 — Fig. S1. Classification of 743 annotated genes from Asian seabass in subcategories of biological process following GO. [file 1471-2164-11-356-S2.DOC]

**Fig. S1 Classification of 743 annotated genes from Asian seabass in subcategories of biological process following GO**
