# Supplementary material for: Identification and analysis of immune-related transcriptome in Asian seabass Lates calcarifer
Source: BMC Genomics. 2010 Jun 4;11:356. doi: 10.1186/1471-2164-11-356 (PMC2893601; doi:10.1186/1471-2164-11-356)
Supplement: Additional file 3 — Fig. S2. Classification of 743 annotated genes from Asian seabass in subcategories of cellular component following GO. [file 1471-2164-11-356-S3.DOC]

**Fig. S2 Classification of 743 annotated genes from Asian seabass in subcategories of cellular component following GO**
