# Supplementary material for: Identification and analysis of immune-related transcriptome in Asian seabass Lates calcarifer
Source: BMC Genomics. 2010 Jun 4;11:356. doi: 10.1186/1471-2164-11-356 (PMC2893601; doi:10.1186/1471-2164-11-356)
Supplement: Additional file 9 — Lca-SSH qRT-PCR MIQE checklist. The qRT-PCR experiment report. [file 1471-2164-11-356-S9.DOC]

| **EXPERIMENTAL DESIGN** |
| --- |
| Definition of experimental and control groups |
| Number within each group |
| Assay carried out by core lab or investigator's lab? |
| Acknowledgement of authors' contributions |
| **SAMPLE** |
| Description |
| Volume/mass of sample processed |
| Microdissection or macrodissection |
| Processing procedure |
| If frozen - how and how quickly? |
| If fixed - with what, how quickly? |
| Sample storage conditions and duration (especially for FFPE samples) |

Experimental design is provided in the material and method section. Total RNA from spleen, liver and kidney of LPS treated seabass (3 fishes) and control (3 fishes) sampled at 24h post challenges were isolated using the TRIZOL kit (Invitrogen, Carlsbad, USA) according to the manufacturer’s instructions. Assays were carried out in the investigator’s lab.

In general, about 50-100 mg of each tissue was sampled and kept in Trizol reagent (Invitrogen, Carlsbad, USA) at -80 ^o^C until RNA isolation within one week.

| **NUCLEIC ACID EXTRACTION** |
| --- |
| Procedure and/or instrumentation |
| Name of kit and details of any modifications |
| Source of additional reagents used |
| Details of DNase or RNAse treatment |
| Contamination assessment (DNA or RNA) |
| Nucleic acid quantification |
| Instrument and method |
| Purity (A260/A280) |
| Yield |
| RNA integrity method/instrument |
| RIN/RQI or Cq of 3' and 5' transcripts |
| Electrophoresis traces |
| Inhibition testing (Cq dilutions, spike or other) |

Total RNA from spleen, kidney and liver were isolated using the TRIZOL kit (Invitrogen, Carlsbad, USA) according to the manufacturer’s instructions. RNA were measured using a NanoDrop Spectrophotometer, ND-1000 (NanoDrop Technologies, Wilmington, USA). The A260/280 ratio was generally between 1.9 and 2.0. However, the yield was significantly different among organs. Exact values of A260/280 ratio for each RNA and yield for each tissue could be provided upon request.

RNA integrity was evaluated by running 1.5 % agrarose gel electrophoresis. To remove any remaining DNA traces, ~ 4µg of the total RNA was treated with 1unit of DNase I recombinant (Roche, Branchburg, USA) in a 10 µl volume according to the manufacturer’s instructions but with 40U RNasin ribonuclease inhibitor (Promega, Madison, USA), and purified by phenol-chloroform after heat inactivation of the enzyme. RNA precipitation was performed finally with ethanol and glycogen overnight at -20 ^o^C. The precipitated RNA was resuspended in water. RNA were measured using a NanoDrop Spectrophotometer, ND-1000 (NanoDrop Technologies, Wilmington, USA).

Contamination was assessed by a no RT control or direct use of treated RNA in the qPCR reaction. Inhibition testing was not performed.

| **REVERSE TRANSCRIPTION** |
| --- |
| Complete reaction conditions |
| Amount of RNA and reaction volume |
| Priming oligonucleotide (if using GSP) and concentration |
| Reverse transcriptase and concentration |
| Temperature and time |
| Manufacturer of reagents and catalogue numbers |
| Cqs with and without RT |
| Storage conditions of cDNA |

Around 1µg aliquot of the DNase-treated total RNA were reverse transcribed to cDNA by M-MLV reverse transcriptase (Promega, Madison, USA) with 0.67 µM poly dT as RT primer in 15 µl volume following the manufacturer's protocol. The reaction mixture of the RNA template and RT primer was heated to 70°C for 5 min to denature the RNA and then incubated on ice for 5 min. The remaining reagents as specified in the thermoscript protocol and 40U RNasin ribo- nuclease inhibitor (Promega, Madison, USA) were added and the reaction was proceeded for 1 hour at 42°C. Finally, the reverse transcriptase was inactivated by 15 min incubation at 70°C. All other steps were performed according manufacturer’s instructions. For most cDNAs, the EF1A amplicon was used for the estimation of contamination and in this case the Ct with RT were about 15-20, without RT no amplification was detected. cDNA was stored at -20°C.

| **qPCR TARGET INFORMATION** |
| --- |
| If multiplex, efficiency and LOD of each assay. |
| Sequence accession number |
| Location of amplicon |
| Amplicon length |
| *In silico* specificity screen (BLAST, etc) |
| Pseudogenes, retropseudogenes or other homologs? |
| Sequence alignment |
| Secondary structure analysis of amplicon |
| Location of each primer by exon or intron (if applicable) |
| What splice variants are targeted? |

Multiplex qPCR was not performed. Gene name and sequence accession numbers were LY6D (GT220983), C1QL4L (GT221611), G0s2 (GT220229), SAA3 (GT221173), NCAN (GT221330), HMOX1(GT219342), DBPHT (GT219284), KRT8 (GT221750), FGG(GT221329), IGHM(GT221130), APOE(GT221763), MYD88(GT219458), CMKLR1(GT219505), Cg0105(GT221495), Cg0526(GT221703), EF1A(GT221873) (note: for the gene that represented by multiple ESTs in libraries the EST accession number containing the lengthiest sequence was provided). Amplicon length was included in supplemental file Table S5. *In silico* screen were performed with NCBI Blast. Primers were designed in exons or UTR regions. Secondary structure analysis of amplicon was not performed. No splice variants were targeted.

| **qPCR OLIGONUCLEOTIDES** |
| --- |
| Primer sequences |
| RT Primer DB Identification Number |
| Probe sequences |
| Location and identity of any modifications |
| Manufacturer of oligonucleotides |
| Purification method |

Primer sequences were included in the manuscript as supplemental file Table S5. No modifications were used. Primers were purchase from 1st BASE Pte Ltd (Singapore) and are salt-free.

| **qPCR PROTOCOL** |
| --- |
| Complete reaction conditions |
| Reaction volume and amount of cDNA/DNA |
| Primer, (probe), Mg++ and dNTP concentrations |
| Polymerase identity and concentration |
| Buffer/kit identity and manufacturer |
| Exact chemical constitution of the buffer |
| Additives (SYBR Green I, DMSO, etc.) |
| Manufacturer of plates/tubes and catalog number |
| Complete thermocycling parameters |
| Reaction setup (manual/robotic) |
| Manufacturer of qPCR instrument |

Each qPCR reaction had a 25 µl reaction volume containing:

iQ SYBR Green Supermix (Bio-Rad, Hercules, CA, USA; cat# 170-8882) 12.5 µl

cDNA (1: 10 dilution of 1^st^ cDNA) 1 µl

200 nM of each forward and reverse primer 0.5 µl

Sterile water 11 µl

96-well PCR plates were purchased from AB Gene (Cat#AB-0600) and adhesive sealer were purchased from Bio-Rad (Cat# MSB1001).

Cycling parameters were:

1 cycle: 95°C for 3 min,

40 cycles: 95°C for 5 sec

60°C for 10 sec

72°C for 20 sec

Melting curve from 60°C to 95°C.

Reactions were set up manually in a designated room using designated equipment. qPCRs were performed with the iQ5 from Bio-Rad.

| **qPCR VALIDATION** |
| --- |
| Evidence of optimisation (from gradients) |
| Specificity (gel, sequence, melt, or digest) |
| For SYBR Green I, Cq of the NTC |
| Standard curves with slope and y-intercept |
| PCR efficiency calculated from slope |
| Confidence interval for PCR efficiency or standard error |
| r2 of standard curve |
| Linear dynamic range |
| Cq variation at lower limit |
| Confidence intervals throughout range |
| Evidence for limit of detection |
| If multiplex, efficiency and LOD of each assay. |

The specificity of the amplification products have been confirmed by size estimations on a 2% agarose gel and by analyzing their melting curves. Without a template, no Cq could be determined since it never passed the threshold line. Standard curves and multiplex PCR were not used in study.

| **DATA ANALYSIS** |
| --- |
| qPCR analysis program (source, version) |
| Cq method determination |
| Outlier identification and disposition |
| Results of NTCs |
| Justification of number and choice of reference genes |
| Description of normalisation method |
| Number and concordance of biological replicates |
| Number and stage (RT or qPCR) of technical replicates |
| Repeatability (intra-assay variation) |
| Reproducibility (inter-assay variation, %CV) |
| Power analysis |
| Statistical methods for result significance |
| Software (source, version) |
| Cq or raw data submission using RDML |

qPCR analysis program (source, version): Bio-Rad, iQ5 (Ver. 2.0)

Cq’s were determined using an analysis Mode of PCR Base Line Subtracted Curve Fit. No data were excluded from the calculations.

Results of NTCs: no amplification products was presented, thus no Cqs.

Justification of number and choice of reference genes: Between tissues, EF1A were most stable and previously was tested in several studies with the same results (e.g., Jorgensen, et al., 2006; Olsvik, et al., 2005).

Description of normalisation method: endogenous reference gene.

Number and concordance of biological replicates: 3

Number and stage (RT or qPCR) of technical replicates: 3 at qPCR level.

Repeatability (intra-assay variation): was below one Cq.

Statistical methods for result significance: For testing the null hypothesis that the two means of gene expressions between LPS treated seabass and control were equal, a two-tailed T-test was performed by using the web calculator ‘Independent groups T-TEST for means calculator’ (http://www.dimensionresearch.com/resources/calculators/ttest.html). A confidence level of 99% was used in the test.

Jorgensen, S.M., Kleveland,E.J., Grimholt,U. and Gjoen, T. Validation of Reference Genes for Real-Time Polymerase Chain Reaction Studies in Atlantic Salmon. [Marine Biotechnology](http://www.springerlink.com/content/103465/?p=c68232646f38478e8e07fced301cdcef&pi=0), 2006, 8(4): 398-408.

Olsvik,P.A., Lie,K.K., Jordal,A.O., Nilsen,T.O., and Hordvik,I. Evaluation of potential reference genes in real-time RT-PCR studies of Atlantic salmon. BMC Molecular Biology, 2005, 6:21
